# Supplementary material for: Cost-Effectiveness of Blood-Based Fibrosis Screening in High-Risk Metabolic Liver Diseases With Emerging Therapies
Source: Gastro Hep Adv. 2026 Mar 13;5(5):100923. doi: 10.1016/j.gastha.2026.100923 (PMC13087396; doi:10.1016/j.gastha.2026.100923)
Supplement: Supplementary Appendix [file mmc1.pdf]

Supplementary Appendix

Cost-effectiveness of blood-based fibrosis screening in high-risk metabolic liver diseases with emerging therapies

Wanyi Chen[1], Stephanie T. Chang[2,3], Ramsey C. Cheung[4,5], Donald B. Chalfin[1,6], Kinpritma Sangha[7], Szu-Yu Zoe Kao[7], Artem T. Boltyenkov[1]

[1] Medical Affairs, Siemens Healthcare Diagnostic Inc., Tarrytown, NY, US

[2] Department of Radiology, Veterans Affairs Palo Alto Healthcare System, Palo Alto, CA, US

[3] Department of Radiology, Stanford University Medical Center, Stanford, CA, US

[4] Department of Gastroenterology and Hepatology, VA Palo Alto Healthcare System, Palo Alto, CA, US

[5] Department of Gastroenterology and Hepatology, Stanford University Medical Center, Stanford, CA, US

[6] Jefferson College of Population Health, Thomas Jefferson University, Philadelphia, PA, US

[7] Siemens Medical Solutions USA Inc., Malvern, PA, US

Supplementary Introduction

In this supplementary appendix, we provide further details of the cost-effectiveness analysis framework. We also provide additional results.

Supplementary Methods

Cost-effectiveness analysis framework

Cost-effectiveness analysis (CEA) is a well-established analytic framework used to compare the costs and health outcomes of one or more health interventions.[49,50] A CEA aims to identify the health intervention(s) that maximize health benefits subject to resource constraints. Here, we provide a brief survey of the key CEA concepts used in our study.

One of the main outcomes of a CEA is the incremental cost-effectiveness ratio (ICER), defined as the ratio of the difference in costs between two alternative strategies to the difference in effectiveness between the same two alternatives. When performing the base case analysis, strategies are first ordered based on increasing costs. Strategies can be eliminated if they result in lower health benefits (e.g., QALYs) and cost more than an alternative (namely, strong dominance). After eliminating strategies based on strong dominance, the remaining strategies are ordered in both increasing costs and health outcomes. To identify the most cost-effective strategy among the remaining contenders, the concept of weak dominance first applies. Consider three strategies of increasing costs and health outcomes: A, B, and C. Strategy B can be eliminated based on weak dominance if the ICER between C and A is lower than the ICER between B and A. Intuitively, this is because both B and C improves health outcomes compared to A, however, C improves outcomes at a lower cost per unit of improvement than B does, due to having lower ICER.

After applying both strong and weak dominance rules, the remaining strategies are said to be on the cost-efficiency frontier. To identify the optimal strategy given budget constraints, we need the concept of willingness-to-pay threshold (WTP), defined as the dollar amount that an individual or society is willing to pay for the additional per unit of health benefit gained.[51] The WTP threshold makes the ICER actionable for decision making, as it is used to judge whether the health gains offered by a health intervention are sufficiently large relative to the costs for the intervention to be adopted. In our study, we adopt a widely adopted WTP threshold of $100,000/QALY, which was empirically estimated based on the concept of health opportunity cost.[52]

Finally, the most cost-effective strategy given a WTP threshold is the strategy associated with the greatest ICER (compared to the next costly strategy) that does not exceed the WTP threshold. The remaining strategies on the cost-efficiency frontier are said to be less cost-effective. Those eliminated by dominance rules are said to be not cost-effective.

Supplementary Results

Appendix Figure A1. Natural history of MASLD model structure.

Appendix Table A1. Additional base case results for a cost-effectiveness analysis of blood-based fibrosis screening in a real-world population with suspected MASLD, for the full population, the subgroup aged ≥65 years, and the lower-risk population with fewer metabolic comorbidities.

Annual NIT-based screening strategies	Life expectancy, years	Number of DCC, cases/10,000	Number of HCC, cases/10,000	Number of LT, Cases/10,000	Number treated with Resmetirom/100
**Full population**					
FIB-4 1.3-2.67/ELF 9.80	12.775	447	297	45.8	32.2
ELF 9.80	12.776	446	297	45.5	32.2
FIB-4 1.3-2.67/ELF 9.00	12.808	437	288	44.2	34.9
FIB-4 1.3	12.809	437	288	44.0	34.9
FIB-4 1.3-2.67/ELF 7.70	12.809	437	288	44.0	34.9
ELF 9.00	12.818	435	286	43.6	35.7
**Subgroup aged ≥65 years**					
FIB-4 1.3-2.67/ELF 9.80	12.654	486	322	48.7	21.2
FIB-4 1.3	12.654	484	323	49.3	21.2
FIB-4 1.3-2.67/ELF 9.00	12.655	485	323	49.2	21.2
FIB-4 1.3-2.67/ELF 7.70	12.654	484	323	49.3	21.2
ELF 9.80	12.786	443	293	44.6	33.3
ELF 9.00	12.818	435	286	43.5	35.7

Appendix Table A1. Continued.

Annual NIT-based screening strategies	Life expectancy, years	Number of DCC, cases/10,000	Number of HCC, cases/10,000	Number of LT, Cases/10,000	Number treated with Resmetirom/100
**Lower-risk population with fewer metabolic comorbidities**					
FIB-4 1.3-2.67/ELF 9.80	17.960	280	312	99.5	24.7
ELF 9.80	17.960	280	311	99.4	24.7
FIB-4 1.3-2.67/ELF 9.00	17.966	275	306	97.5	25.8
FIB-4 1.3	17.967	275	306	97.5	25.8
FIB-4 1.3-2.67/ELF 7.70	17.967	275	306	97.5	25.8
ELF 9.00	17.970	274	305	97.1	26.1

Abbreviations: DCC, decompensated cirrhosis; ELF, enhanced liver fibrosis; FIB-4, fibrosis index; HCC, hepatocellular carcinoma; LT, liver transplantation.

CHEERS checklist

	Item	Guidance for Reporting	Reported in section
**TITLE**			
Title	1	Identify the study as an economic evaluation and specify the interventions being compared.	Page 1
**ABSTRACT**			
Abstract	2	Provide a structured summary that highlights context, key methods, results and alternative analyses.	Page 4
**INTRODUCTION**			
Background and objectives	3	Give the context for the study, the study question and its practical relevance for decision making in policy or practice.	Page 5-6
**METHODS**			
Health economic analysis plan	4	Indicate whether a health economic analysis plan was developed and where available.	Page 7-8
Study population	5	Describe characteristics of the study population (such as age range, demographics, socioeconomic, or clinical characteristics).	Page 9
Setting and location	6	Provide relevant contextual information that may influence findings.	Page 7-9
Comparators	7	Describe the interventions or strategies being compared and why chosen.	Page 7
Perspective	8	State the perspective(s) adopted by the study and why chosen.	Page 8
Time horizon	9	State the time horizon for the study and why appropriate.	Page 8
Discount rate	10	Report the discount rate(s) and reason chosen.	Page 8
Selection of outcomes	11	Describe what outcomes were used as the measure(s) of benefit(s) and harm(s).	Page 7-8
Measurement of outcomes	12	Describe how outcomes used to capture benefit(s) and harm(s) were measured.	Page 7-8
Valuation of outcomes	13	Describe the population and methods used to measure and value outcomes.	Page 7-10
Measurement and valuation of resources and costs	14	Describe how costs were valued.	Page 8, 10
Currency, price date, and conversion	15	Report the dates of the estimated resource quantities and unit costs, plus the currency and year of conversion.	Page 8
Rationale and description of model	16	If modelling is used, describe in detail and why used. Report if the model is publicly available and where it can be accessed.	Page 8-9
Analytics and assumptions	17	Describe any methods for analysing or statistically transforming data, any extrapolation methods, and approaches for validating any model used.	NA

Characterizing heterogeneity	18	Describe any methods used for estimating how the results of the study vary for sub-groups.	Page 6-7
Characterizing distributional effects	19	Describe how impacts are distributed across different individuals or adjustments made to reflect priority populations.	NA
Characterizing uncertainty	20	Describe methods to characterize any sources of uncertainty in the analysis.	Page 9-11
Approach to engagement with patients and others affected by the study	21	Describe any approaches to engage patients or service recipients, the general public, communities, or stakeholders (e.g., clinicians or payers) in the design of the study.	NA
**RESULTS**			
Study parameters	22	Report all analytic inputs (e.g., values, ranges, references) including uncertainty or distributional assumptions.	Page 9-10, Table 1 and 2
Summary of main results	23	Report the mean values for the main categories of costs and outcomes of interest and summarise them in the most appropriate overall measure.	Page 12-14, Table 3, Figure 1-3
Effect of uncertainty	24	Describe how uncertainty about analytic judgments, inputs, or projections affect findings. Report the effect of choice of discount rate and time horizon, if applicable.	Page 13-14, Figure 2-3
Effect of engagement with patients and others affected by the study	25	Report on any difference patient/service recipient, general public, community, or stakeholder involvement made to the approach or findings of the study	NA
**DISCUSSION**			
Study findings, limitations, generalizability, and current knowledge	26	Report key findings, limitations, ethical or equity considerations not captured, and how these could impact patients, policy, or practice.	Page 15-17
**OTHER RELEVANT INFORMATION**			
Source of funding	27	Describe how the study was funded and any role of the funder in the identification, design, conduct, and reporting of the analysis	Page 2
Conflicts of interest	28	Report authors conflicts of interest according to journal or International Committee of Medical Journal Editors requirements.	Page 2
