# Supplementary figures and images for: Cost-Effectiveness of Blood-Based Fibrosis Screening in High-Risk Metabolic Liver Diseases With Emerging Therapies
Source: Gastro Hep Adv. 2026 Mar 13;5(5):100923. doi: 10.1016/j.gastha.2026.100923 (PMC13087396; doi:10.1016/j.gastha.2026.100923)

# Natural history model

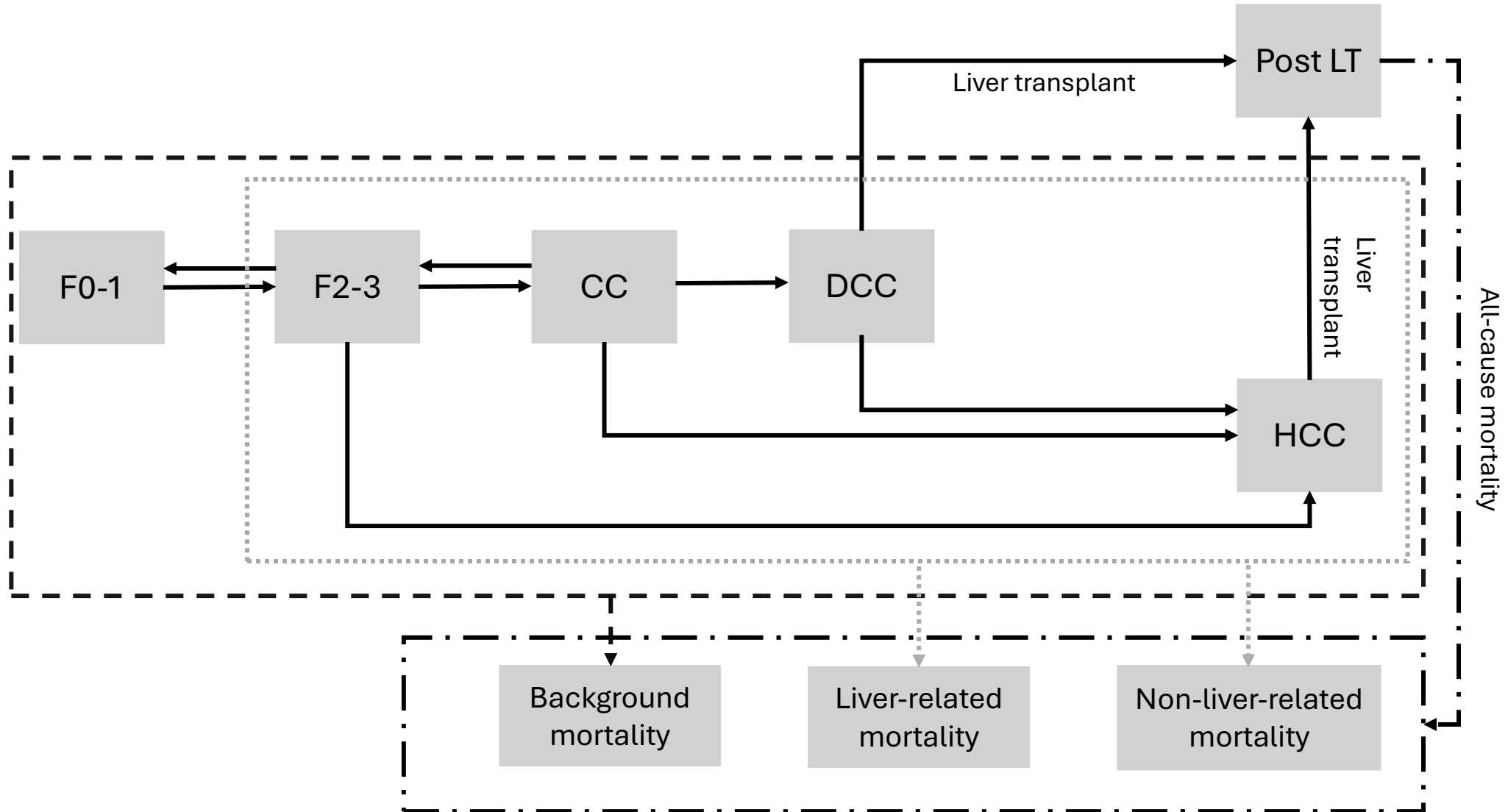

Supplement: Supplementary Figure [file mmc2.pdf]
