# Supplementary material for: Cost-Effectiveness of Blood-Based Fibrosis Screening in High-Risk Metabolic Liver Diseases With Emerging Therapies
Source: Gastro Hep Adv. 2026 Mar 13;5(5):100923. doi: 10.1016/j.gastha.2026.100923 (PMC13087396; doi:10.1016/j.gastha.2026.100923)

Gastro Hep Advances 2026;5:100923

ORIGINAL RESEARCH—CLINICAL

Cost-Effectiveness of Blood-Based Fibrosis Screening in High-Risk Metabolic Liver Diseases With Emerging Therapies

Wanyi Chen,[1] Stephanie T. Chang,[2,3] Ramsey C. Cheung,[4,5] Donald B. Chalfin,[1,6] Kinpritma Sangha,[7] Szu-Yu Zoe Kao,[7] and Artem T. Boltyenkov[1]

[1]Medical Affairs, Siemens Healthcare Diagnostics Inc., Tarrytown, New York; [2]Department of Radiology, Veterans Affairs Palo Alto Healthcare System, Palo Alto, California; [3]Department of Radiology, Stanford University Medical Center, Stanford, California; [4]Department of Gastroenterology and Hepatology, VA Palo Alto Healthcare System, Palo Alto, California; [5]Department of Gastroenterology and Hepatology, Stanford University Medical Center, Stanford, California; [6]Department of Nursing, Jefferson College of Population Health, Thomas Jefferson University, Philadelphia, Pennsylvania; and [7]Research, Innovation and Scientific Engagement (RISE), Siemens Medical Solutions USA Inc., Malvern, Pennsylvania

BACKGROUND AND AIMS: Metabolic dysfunction–associated steatotic liver disease (MASLD) remains a global public health threat. With emerging effective pharmacologic therapies, early identification of significant fibrosis in primary care is critical. We evaluated the cost-effectiveness of blood-based noninvasive tests incorporating real-world diagnostic performance in the contemporary MASLD treatment era. **METHODS:** We developed a decision-analytic model for MASLD natural history and treatment to compare 6 noninvasive test strategies using Fibrosis-4 (FIB-4) and/or enhanced liver fibrosis (ELF) tests at varying cutoffs. Patients identified with significant fibrosis were referred for hepatology staging (biopsy and/or imaging-based), and eligible individuals were considered for Resmetirom ($47,400/y; mean duration: 1.6–1.8 year). Model parameters were informed by a real-world cohort (n = 400) of high-risk primary care patients with suspected MASLD who underwent both tests. Outcomes included quality-adjusted life-years (QALYs), lifetime costs, incremental cost-effectiveness ratios, and adverse liver outcomes averted. **RESULTS:** The modeled population had a mean age of 64 years, mean body mass index of 32 kg/m^2, with 82% having type-2 diabetes and 15% significant fibrosis. Sequential screening with ELF (cutoff 9.80) following indeterminate FIB-4 (1.3–2.67) was the most cost-effective, yielding 8.610 QALYs and $96,990 in lifetime costs at a $100,000/QALY threshold. ELF-alone screening at a 9.00 cutoff maximized QALYs and individuals treated but increased unnecessary referrals. Resmetirom cost was most influential on results: if cost fell below $11,570/y (base case, $47,400/y), ELF-alone screening became the preferred strategy. Findings remained robust across sensitivity analyses, including in low-risk populations. **CONCLUSION:** By integrating real-world diagnostic performance with new MASLD therapies, this translational modeling study identifies a scalable, cost-effective fibrosis screening pathway using FIB-4 and ELF. These findings support implementation of blood-based fibrosis screening in general primary care populations.

Keywords: MASLD; Noninvasive Tests (NITs); ELF; Resmetirom; Economic Evaluation

Introduction

Global prevalence of metabolic dysfunction–associated steatotic liver disease (MASLD) is projected to reach 56% by 2040.[1] In the United States, MASLD is a leading cause of cirrhosis, liver cancer, and liver transplantation,[2] incurring $1.6 billion annually in direct medical costs.[3,4] MASLD encompasses a spectrum of diseases, including metabolic dysfunction–associated steatohepatitis (MASH) and cirrhosis. Type 2 diabetes (T2D) and obesity are tightly linked to MASLD disease progression[5] with a hazard ratio of 1.4–2.6 compared to those without these metabolic risk factors.[6,7] Three-quarters of individuals with obesity and 69% of individuals with T2D have MASLD.[8]

Intervention at an early stage of fibrosis is imperative to prevent future complications, namely cirrhosis, decompensation, and liver cancer.[9] Noninvasive tests (NITs), including blood-based markers (eg, Fibrosis-4 [FIB-4] and enhanced liver fibrosis [ELF][10]), and imaging-based tests (eg, vibration-controlled transient elastography [TE], magnetic resonance elastography [MRE]), comprise some of the diagnostic tools for fibrosis assessment.[11,12] Globally, there is growing recognition that readily available laboratory data upstream of primary care, such as elevated alanine aminotransferase (ALT > 30 U/L), can serve as automated triggers to warrant further primary care-based fibrosis risk

Abbreviations used in this paper: **ALT, aminotransferase; DCC, decompensated cirrhosis; ELF, enhanced liver fibrosis; FIB-4, fibrosis-4; HCC, hepatocellular carcinoma; ICER, incremental cost-effectiveness ratio; LE, life expectancy; LT, liver transplant; MASH, metabolic dysfunction–associated steatohepatitis; MASLD, metabolic dysfunction–associated steatotic liver disease; MRE, magnetic resonance elastography; NIT, noninvasive test; QALYs, quality-adjusted life-years; RR, relative risk; T2D, type 2 diabetes; TE, transient elastography.**

Most current article

© 2026 The Authors. Published by Elsevier Inc. on behalf of American Gastroenterological Association Institute. This is an open access article under the CC BY license (http://creativecommons.org/licenses/by/4.0/).
2772-5723
https://doi.org/10.1016/j.gastha.2026.100923

assessment.[13–15] Meanwhile, noninvasive imaging-based fibrosis assessment downstream of primary care, particularly TE, has gained increasing real-world application in specialty care settings, reflecting expanding capacity and demand for fibrosis staging.[16–18] At the moment, ELF is the only authorized commercial test in the United States for advanced fibrosis due to MASH, demonstrating high prognostic value.[19] Major liver societies recommend a stepwise approach using FIB-4 followed by ELF or TE for risk stratification in primary care.[20,21]

The therapeutic landscape of MASLD has shifted rapidly. In 2024, Resmetirom became the first FDA-approved pharmacological treatment for MASH.[22] Several other promising agents are in advanced stages of development, including Semagultide (Wegovy), which just received FDA approval.[23,24] With emerging effective treatments, early identification of at-risk individuals is increasingly critical. Due to a strong association with T2D and obesity, MASLD is often first encountered in primary care or endocrine clinics.[25] Although guidelines advocate NIT-based risk stratification in these settings, utilization remains low due to limited provider awareness, unclear care pathways after diagnosis, concerns of over referral, and uncertainty about cost-effectiveness.[20,25,26]

Prior cost-effectiveness analyses have supported sequential FIB-4/ELF screening for reducing unnecessary referrals[27–29]; however, long-term results vary due to differing assumptions about the diagnosis impact on treatment.[30–32] Only 1 study, to our knowledge, incorporated Resmetirom as the downstream management after identification of significant fibrosis[32]; however, real-world diagnostic performance data were not directly linked to therapeutic decision-making.

To bridge this translational gap, we integrated real-world diagnostic accuracy of blood-based NITs with contemporary Resmetirom treatment to evaluate the cost-effectiveness of fibrosis screening strategies. Given limited TE availability in primary care and the absence of consensus on optimal screening approaches, we sought to identify the most cost-effective NITs using FIB-4 and/or ELF. Our modeled cohort was informed by an empiric population with high prevalence of T2D and obesity, representing a high-risk group commonly seen in clinical practice. Furthermore, we focused on Resmetirom impact as it is currently the only MASH-targeting therapy with published instructions for use. This analysis thus provides evidence that is broadly generalizable to high-risk primary care populations and offer practical guidance for implementing cost-effective, blood-based fibrosis screening in the evolving MASLD therapeutic landscape.

Methods

Analytic Overview

We developed an individual-level state-transition model to evaluate the long-term clinical and economic impact of NITs for identifying significant fibrosis in a high-risk, primary care population informed by a cohort with high prevalence of T2D and/or obesity. We modeled the following 6 annual NITs, using FIB-4 and ELF, individually or combined, with guideline-based cutoffs (Figure 1)[20,21,33]:

1) FIB-4 (cutoff 1.3, “FIB-4 1.3”)
2) ELF (cutoff 9.00, “ELF 9.00”)
3) ELF (cutoff 9.80, “ELF 9.80”)
4) Indeterminate FIB-4 (1.3–2.67) followed by ELF (cutoff 7.70, “FIB-4 1.3–2.67/ELF 7.70”)
5) “FIB-4 1.3–2.67/ELF 9.00”
6) “FIB-4 1.3–2.67/ELF 9.80”

In strategies 1–3 above, only patients with a single test result above the cutoff will be referred to hepatology (Figure 1). In sequential testing, strategies 4–6, patients with FIB-4 score $<$ 1.3 are classified as having nonsignificant fibrosis and managed by primary care, patients with FIB-4 score $>$ 2.67 are classified as significant fibrosis and referred to hepatology, while patients with FIB-4 score between 1.3 and 2.67 receive an additional ELF test that determines hepatology referral (Figure 1). We did not include no screening as NIT-based screening among high-risk populations were shown to be cost-effective compared to no screening and was recommended by multiple guidelines.[20,21,31,32] Regardless of the strategy, those who are referred to hepatology will be correctly staged using liver biopsy, ELF, or imaging-based tools (TE, MRE, or liver ultrasound). Patients identified with stage 2 or 3 MASH will be offered Resmetriom with imperfect uptake and persistence, and patients identified with nonsignificant fibrosis will be sent back to primary care and retested in 1–2 years. Those who are not referred to hepatology are offered the same NIT strategy annually. Screening uptake is imperfect. For each NIT, we projected outcomes for a lifetime horizon: number of decompensated cirrhosis (DCC), hepatocellular carcinoma (HCC), liver transplant (LT) cases, undiscounted life expectancy (LE), discounted quality-adjusted life-years (QALYs), discounted costs, and incremental cost-effectiveness ratios (ICERs) in 2025 United States dollars per QALY gained. We applied an annual rate of 3% when discounting QALYs and costs. We used a health-care payer perspective,[34] including the costs of NITs, hepatology workup (visit and staging), and downstream care costs for clinical management of various MASLD disease stages. The most cost-effective NIT(s) is defined as having the greatest ICER below a willingness-to-pay threshold of \$100,000/QALY. We provide a more detailed description of the cost-effectiveness analysis framework in Supplementary Appendix, Method. Our model was built using TreeAge Pro 2021 (Williamstown, MA, www.treeage.com). Institutional review board approval was not necessary due to no real-patient records being involved in the modeling. The reporting of this study followed the Consolidated Health Economic Evaluation Reporting Standards,[35] with a compliance checklist provided in Supplementary Appendix.

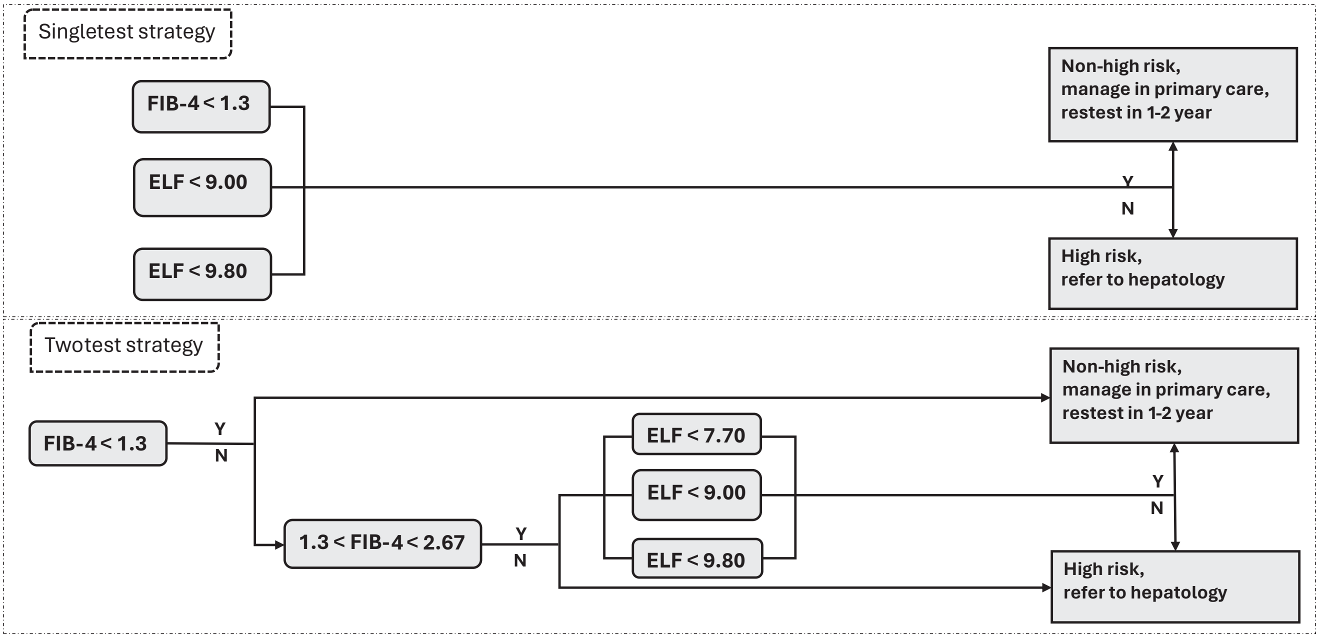

Figure 1. Modeled NIT-based screening strategies among a real-world population with suspected MASLD, from top to bottom: “FIB-4 1.3”; “ELF 9.00”; “ELF 9.80”; “FIB-4 1.3–2.67/ELF 7.70”; “FIB-4 1.3–2.67/ELF 9.00”; “FIB-4 1.3–2.67/ELF 9.80”.

MASLD Natural History

Our model captured the MASLD natural disease spectrum across 7 stages with increasing severity: MASLD/MASH fibrosis stage 0-1 (F0-1), MASH fibrosis stage 2-3 (F2-3), compensated cirrhosis (CC), decompensated cirrhosis (DCC), hepatocellular carcinoma (HCC), post-LT, and death (Supplementary Figure). We used a 1-year cycle length due to slow progression of MASLD. Within each cycle, patients in states F0-1, F2-3, or CC can progress or regress by 1 stage or remain stable. DCC is considered irreversible. Patients in F2-3, CC, or DCC can develop HCC, with increasing risk at later stages. Those in DCC or HCC may receive LT, transitioning to the post-LT state. All patients are subject to background mortalities based on life tables.[36] Patients in F2-3, CC, DCC, and HCC are subject to both liver- and non–liver-related (ie, cardiovascular and nonhepatic neoplasm–related) mortalities. Those in F0-1 are only subject to non–liver-related mortalities. Mortality in post-LT state is modeled as time-varying all-cause mortality based on registry data.[37]

MASLD Treatment

Resmetirom, FDA-approved in 2024 following a phase 3 trial, improves fibrosis regression and reduces progression in noncirrhotic MASH (F2-3).[38] We quantify treatment effect using the concept of relative risk (RR), defined as the ratio of fibrosis progression/regression probabilities with treatment to those without treatment. An RR $>$ 1 of fibrosis regression with treatment indicates that treatment is effective in improving fibrosis; while a RR $<$ 1 of fibrosis progression with treatment suggests that treatment is effective in slowing down progression. Specifically, we assume that treatment increases the likelihood of fibrosis regression from state F2-3 to F0-1 and decreases the likelihood of fibrosis progression from state F2-3 to CC, DCC, and HCC. To reflect real-world suboptimal compliance with Resmetirom, we model imperfect initial uptake and subsequent discontinuation (due to side effects) for those offered the treatment option.[39,40]

Model Inputs

Cohort characteristics. We simulated a 1-million cohort representative of the participants from a cross-sectional study, which prospectively recruited 400 people with T2D and/or obesity from primary care clinics in the Veteran Affairs Palo Alto Healthcare System (VAPAHCS, Palo Alto, CA), from May 2022 to June 2024 (Siemens Healthineers and VA Palo Alto, unpublished data, 5/2022-10/2023).[29] All study participants underwent a physical exam and lab testing using FIB-4 and ELF. Mean age of the study cohort was 64 years (61%, $\geq$65 years), mean body mass index 32 (standard deviation = 6), and 82% had T2D. FIB-4 distributions were $<$1.3 (62%), 1.3–2.67 (34%), and $\geq$2.67 (4%); ELF distributions were 7.70–9.00 (16%), 9.00–9.80 (34%), and $\geq$9.80 (50%).

Test characteristics. MRE was obtained from a subset (n = 101) of the participants. We derived test accuracy (ie, sensitivity and specificity) for 6 investigated NIT strategies using MRE results as the gold standard for adjudicating true disease state: F2-3 (MRE $\geq$ 3.14 kPa) and F0-1 (MRE $<$ 3.14 kPa), where a cutoff value of 3.14 kPa was recommended by recent guidelines.[20] The resulting diagnostic accuracy is shown in Table 1.

Table 1. Test Characteristics for 6 NIT Strategies Using MRE as the Adjudicator for True Significant Fibrosis for a Real-World Population With Suspected MASLD, Both the Full Population and the Subgroup Aged ≥65 Years

Annual NIT-based screening strategies	Sensitivity[a], %		Specificity[a], %	
	Full population	Subgroup aged ≥65 y	Full population	Subgroup aged ≥65 y
FIB-4 1.3–2.67/ELF 9.80	73	30	43	81
ELF 9.80	73	80	38	29
FIB-4 1.3–2.67/ELF 9.00	93	30	24	83
FIB-4 1.3–2.67/ELF 7.70	93	30	20	86
FIB-4 1.3	93	30	20	81
ELF 9.00	100	100	12	7

[a]Sensitivity and specificity were derived using MRE, as the adjudicator for true disease state based on a subset of 101 patients. A cutoff of 3.14 kPa was used for state F2-3, or significant fibrosis.

Natural history transitions. We derived probabilities of progression, regression, and cause-specific mortalities by averaging estimates from multiple published sources (Table 2).[9,32,37,40,41–46] When available, we used estimates that were based on systematic review or large observational studies (including the Global NASH Registry) of people with T2D or obesity at elevated risk of MASLD as our base case values. This approach reflects the higher baseline progression risks expected in populations with a high prevalence of metabolic comorbidities.

Treatment effect. The phase 3 trial suggested that 24% from the 80-mg Resmetirom arm had fibrosis improvement over 1 year (14%, placebo).[40] Based on this result, we derived an RR of 1.82 (24/14) as the treatment effect on fibrosis regression compared to no treatment. We estimated the RR of fibrosis progression to be 0.56, by using the difference in percentage of participants with a 25% or greater increase in liver stiffness between the 2 arms from the same trial.

Costs and utilities. Our study was conducted from a health-care payer perspective and included direct medical costs associated with each health state (Table 2), including inpatient and outpatient, medications, and procedures. Hepatology workup costs comprised of specialist consultation and fibrosis staging, which reflected standard practice using a combination of liver biopsy, ELF, and imaging-based modalities (TE, MRE, or liver ultrasound). Resource use frequencies for each staging component were estimated from the same real-world cohort and are reported in Table 2. Resmetirom treatment costs were obtained from Centers for Medicare & Medicaid Services ($47,400/y at the time of analysis) and applied exclusively to individuals with F2-3 fibrosis. All costs were sourced from published literature using a combination of micro-costing and gross-costing methods, adjusted using the Consumer Price Index, and reported in 2025 United States dollars. We sourced health utilities scores from published literature using preference-based utility scores.

Sensitivity Analysis

We assessed the impact of parameter uncertainty on the comparative cost-effectiveness of strategies.[49] We used probabilistic sensitivity analysis to gauge decision uncertainty, where all model parameters were varied across evidence-based ranges according to probability distributions. In one-way sensitivity analysis, we focused on 2 strategies identified as most likely to be cost-effective in probabilistic sensitivity analysis and examined the impact of varying individual model parameters on the ICER between the 2 strategies. Key inputs varied were as follows: MASLD disease natural history–related inputs (disease progression/regression from significant/advanced fibrosis), initial prevalence of significant fibrosis, as well as treatment-related inputs, which included the annual cost of Resmetirom, treatment efficacy as measured by RR of fibrosis progression/regression with Resmetirom, and uptake and discontinuation rate of Resmetirom which affected treatment duration. For each input, we used the highest and lowest values found in the literature as sensitivity analyses ranges.

Subgroup Analysis

Older age was suggested as a confounding factor for the diagnostic accuracy of NITs.[50] We performed a subgroup analysis of patients ≥65 years, using 2.0 as the lower cutoff for FIB-4. The diagnostic accuracy of NITs associated with the subgroup of patients ≥65 years is shown in Table 1.

Scenario Analysis

Our base-case cohort was characterized by a high prevalence of T2D or obesity. To investigate the generalizability of results to a lower-risk primary care population with suspected MASLD, we conducted a scenario analysis representing individuals with fewer metabolic comorbidities. Lower prevalence of T2D or obesity was implemented in the model by applying reduced progression and mortality risks. This approach was chosen to reflect the

Table 2. Key Model Inputs for a Cost-Effectiveness Analysis of Blood-Based Fibrosis Screening in a Real-World Population With Suspected MASLD

Parameter	Base value	Range	Source
Natural history transition probability from state F0-1, %/y			
F0-1 to F2-3	6.3	1.3–9.6	32,41
Non–liver-related death	1.6	1.3–1.8	32
Natural history transition probabilities from state F2-3, %/y			
F2-3 to F0-1	2.7	0.9–6	32
F2-3 to CC	9.5	5.0–19	32,41,42
F2-3 to HCC	0.3	0.1–0.5	32,41,43
Liver-related death	3.3	2.8–5.2	32
Non–liver-related death	3.3	2.9–4.6	32
Natural history transition probabilities from state CC, %/y			
CC to F2-3	5.4	4.1–14	32,40
CC to DCC	5.7	3.7–7.7	32,41,42
CC to HCC	2.4	0.1–4.6	32,41,44
Liver-related death	7.3	±20%	32
Non–liver-related death	6.2	±20%	32
Natural history transition probabilities from DCC, %/y			
DCC to HCC	2.4	0.7–4.0	32,41
Liver transplantation from DCC	1.8	0.7–2.9	9,32
Liver-related death	22	8.4–24	9,32,41
Non–liver-related death	4.8	0.8–13	9,32,41
Natural history transition probabilities from HCC, %/y			
Liver transplantation from HCC	3.8	2.7–5.0	9,32
Liver-related death	35	26–55	9,32,41
Non–liver-related death	6.0	0.0–12	9,32
Post liver transplantation, all-cause mortalities, %/y			
Year 1–2	6.8	–	37,45
Year 3–4	12.7	–	37,45
Year 5–9	19.8	–	37,45
Year 10+	35.7	–	37,45
Treatment effect, relative risk (RR) of Resmetirom vs placebo			
RR of fibrosis regression	1.82	1.02–2.89	40,46
RR of fibrosis progression	0.56	0.38–0.96	40,46
Behavior characteristics			
Resmetirom uptake, %	75	20–100	39
Resmetirom discontinuation, %/y	10	0–20	39
Annual screening uptake, %	50	0–100	Assumption
Direct medical cost in various health states, 2025 USD/y			
F0-1	500	480–510	31,41
F2-3	1180	640–1720	41,42
CC	23,360	18,210–28,510	32,41,47
DCC	36,290	30,190–42,390	41,42
HCC[a]	85,840	60,900–110,790	32,41,47
Post liver-transplantation	7630	0–15,250	32,42
Resmetirom	47,400	10,000–90,000	48
One-time cost, 2025 USD			
Liver transplantation	419,000	416,200–421,860	32,41
Liver-related death	46,590	–	32
ELF test	200	170–230	29,32
FIB-4 test	0	–	–
Hepatology workup			
	**Cost, $**	**Resource use, %**	**Source**
Hepatology consultation	257	100	29
ELF test	185	25	
TE	32	25	
Ultrasound liver	136	200	
Endoscopy	367	50	
MRI/CT abdomen/liver	323	5	
Liver biopsy	354	15	

Table 2. Continued

	Cost, $	Resource use, %	Source
Health utilities			
F0-1	0.88	0.84–0.91	31,32,41
F2-3	0.76	0.68–0.84	32,42
CC	0.74	0.68–0.81	32,42
DCC	0.57	0.52–0.63	32,42
HCC	0.5	0.45–0.55	42
Post liver transplantation	0.825	0.81–0.84	32

CT, computed tomography; MRI, magnetic resonance imaging; USD, United States dollar.
[a]Annual care cost in state HCC includes hospitalization and partial utilization of resection, radiotherapy, and radiofrequency ablation.

aggregate effect of metabolic risk factors on disease trajectory without explicitly characterizing individual's comorbidity status, consistent with prior modeling studies.[31,32] Specifically, studies have reported that T2D or obesity increases fibrosis progression risk by a RR up to 1.8[6,25,32]; we thus scaled down base-case progression rates from early to advanced stages by the inverse of this RR. Additionally, recognizing higher mortality risks in MASLD patients with T2D, we adjusted background, liver-, and non–liver-related mortality rates similarly using the inverses of published RRs of 2.14, 22.8, and 3.25, respectively.[32]

Results

Diagnostic Accuracy

Across populations, 2-tier NITs with confirmatory ELF at high cutoffs had lower sensitivity and higher specificity than single-tier NITs using FIB-4 or ELF at low cutoffs (Table 1). In older patients, FIB-4-based NITs showed reduced sensitivity and increased specificity, suggesting age confounded its accuracy. The strategy "FIB-4 1.3–2.67/ELF 9.80" minimized referrals (lowest sensitivity, highest specificity), whereas "ELF 9.00" identified all treatment-eligible patients (100% sensitivity) but generated most referrals (lowest specificity).

Base Case

Clinical. Clinical outcomes improved with strategies of higher sensitivity and lower specificity. Annual screening using "FIB-4 1.3–2.67/ELF 9.80" resulted in 8.610 QALYs (Table 3), or 12.775 undiscounted LYs (Supplementary Table). This increased to a maximum of 8.635 QALYs (12.818 LYs) with "ELF 9.00". Number treated with Resmetirom ranged from 32.2 with "FIB-4 1.3–2.67/ELF 9.80" to 35.7 with "ELF 9.00" (per 100) (Supplementary Table). Number of adverse outcomes was lowest with "ELF 9.00" (435–447 DCC, 286–297 HCC, and 43.6–45.8 LT per 10,000).

Cost and cost-effectiveness. Lifetime costs ranged from $96,990 to $105,560, with highest cost for "ELF 9.00" due to treating most with Resmetirom and rendering longest LE (Table 3). Lifetime spendings on Resmetirom at $47,400/y (Total: $57,540–$65,500; mean duration: 1.6–1.8 year) accounted for most of the lifetime costs. Three strategies were cost-effective, with increasing costs: "FIB-4 1.3–2.67/ELF 9.80," "FIB-4 1.3," and "ELF 9.00." The ICERs were $301,270/QALY ("FIB-4 1.3" vs "FIB-4 1.3–2.67/ELF9.80"), and $457,620/QALY ("ELF 9.00" vs "FIB-4 1.3"). Given a willingness-to-pay threshold of $100,000/QALY, the optimal strategy was "FIB-4 1.3–2.67/ELF 9.80."

Subgroup Analysis

The clinical, cost, and cost-effectiveness outcomes for the subgroup analysis of patients ≥65 years are shown in Table 3 and Supplementary Table. LE ranged from 8.538 to 8.635 QALYs. Total lifetime costs ranged from $74,300 to $106,040. As in the base case, the strategies "FIB-4 1.3–2.67/ELF 9.80" and "ELF 9.00" remained cost-effective, while "FIB-4 1.3" did not. Annual screening using "FIB-4 1.3–2.67/ELF 9.80" remained the most cost-effective strategy at $100,000/QALY for this older subgroup.

Scenario Analysis

For a cohort with lower prevalence of T2D or obesity, projected LE increased to 11.316–11.326 QALYs (Table 3 and Supplementary Table). Total lifetime costs also increased correspondingly and ranged from $114,890 to $122,260. At $100,000/QALY, strategy dominance remained the same as base case, with "FIB-4 1.3–2.67/ELF 9.80" being the most cost-effective strategy and increased ICERs between strategies on the cost-efficiency frontier.

Probability Sensitivity Analysis

In probability sensitivity analysis, the optimal base case strategy, "FIB-4 1.3–2.67/ELF 9.80", remained most likely (≥60% of simulations) to be cost-effective for both the full population and those aged ≥65 years at willingness-to-pay thresholds of $0–$160,000/QALY (Figures 2 and 3). With higher thresholds, "ELF 9.00" became likely cost-effective in

Table 3. Base Case Results for a Cost-Effectiveness Analysis of Blood-Based Fibrosis Screening in a Real-World Population With Suspected MASLD, for the Full Population, the Subgroup Aged ≥65 Y, and the Lower-Risk Population With Fewer Metabolic Comorbidities

Annual NIT-based screening strategies	QALYs, y	Screening cost, $	Hepatology workup cost	Resmetirom cost, $	Total cost, $	ICER, $/QALY
Full population						
FIB-4 1.3–2.67/ELF 9.80	8.610	650	3200	57,540	96,990	–
FIB-4 1.3	8.630	0	4290	63,670	102,960	301,270
ELF 9.00	8.635	860	4390	65,500	105,560	457,620
Dominated[a]						
ELF 9.80	8.610	890	3570	57,530	97,570	–
FIB-4 1.3–2.67/ELF 9.00	8.630	640	3970	63,670	103,270	–
FIB-4 1.3–2.67/ELF 7.70	8.630	640	4290	63,670	103,600	–
Subgroup aged ≥65 y						
FIB-4 1.3–2.67/ELF 9.80	8.538	140	980	35,420	74,300	–
ELF 9.00	8.635	860	4900	65,490	106,040	327,660
Dominated						
FIB-4 1.3	8.538	0	1310	35,400	74,480	–
FIB-4 1.3–2.67/ELF 9.00	8.538	140	1170	35,410	74,480	–
FIB-4 1.3–2.67/ELF 7.70	8.538	140	1310	35,400	74,610	–
ELF 9.80	8.616	880	3860	59,880	99,940	–
Lower-risk population with fewer metabolic comorbidities						
FIB-4 1.3–2.67/ELF 9.80	11.317	810	3910	70,210	114,890	–
FIB-4 1.3	11.324	0	5300	75,270	119,820	747,150
ELF 9.00	11.326	1070	5420	76,660	122,260	1,162,270
Dominated[a]						
ELF 9.80	11.316	1090	4390	70,190	115,620	–
FIB-4 1.3–2.67/ELF 9.00	11.323	790	4880	75,270	120,180	–
FIB-4 1.3–2.67/ELF 7.70	11.324	790	5300	75,270	120,610	–

ELF, enhanced liver fibrosis; FIB-4, fibrosis index; ICER, incremental cost-effectiveness ratio; QALY, quality-adjusted life-years.
[a]A strategy is said to be a dominating strategy, if it is on the cost-efficiency frontier. That is, there exists some willingness-to-pay threshold such that this strategy would be the most cost-effective strategy. Otherwise, a strategy is said to be dominated.

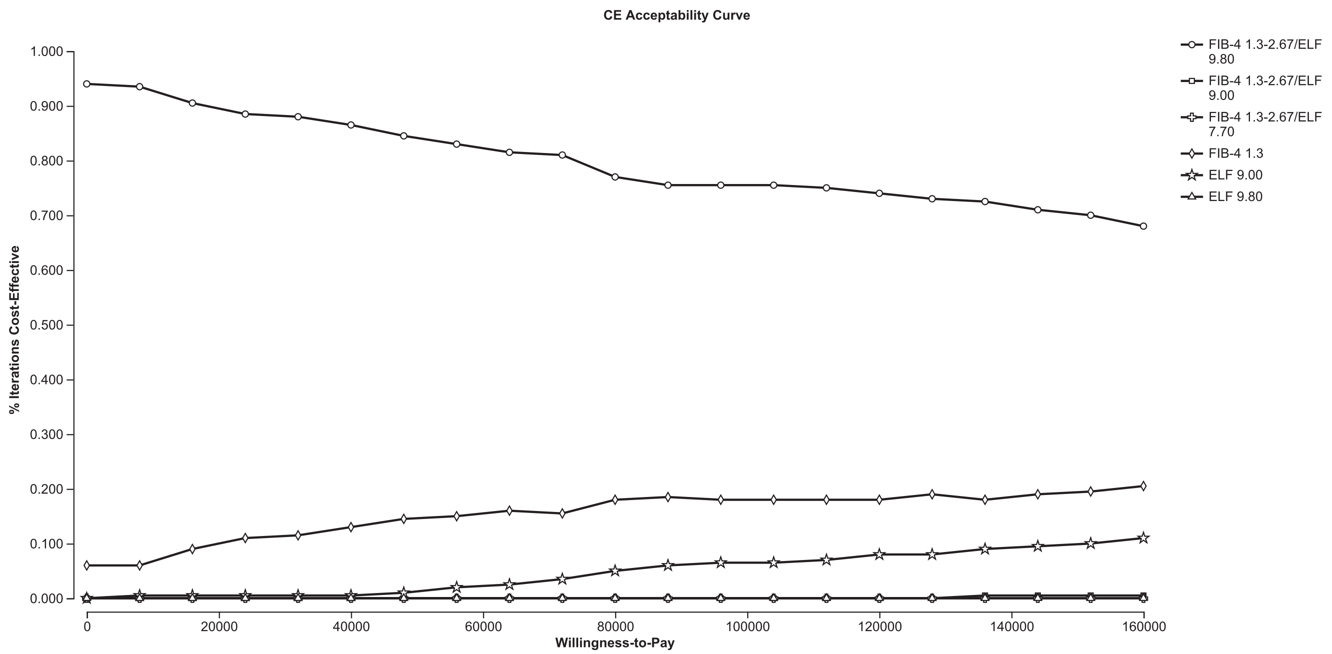

Figure 2. Probability sensitivity analysis for a cost-effectiveness analysis of blood-based fibrosis screening in a real-world population with suspected MASLD: the full population. This plot displays, for the full population, the probability that each strategy was the most cost-effective (y-axis) as willingness-to-pay (x-axis) increased from \$0 to \$160,000/QALY. ELF, enhanced liver fibrosis; FIB-4, fibrosis-4; MASLD, metabolic dysfunction–associated steatotic liver disease; NIT, noninvasive test.

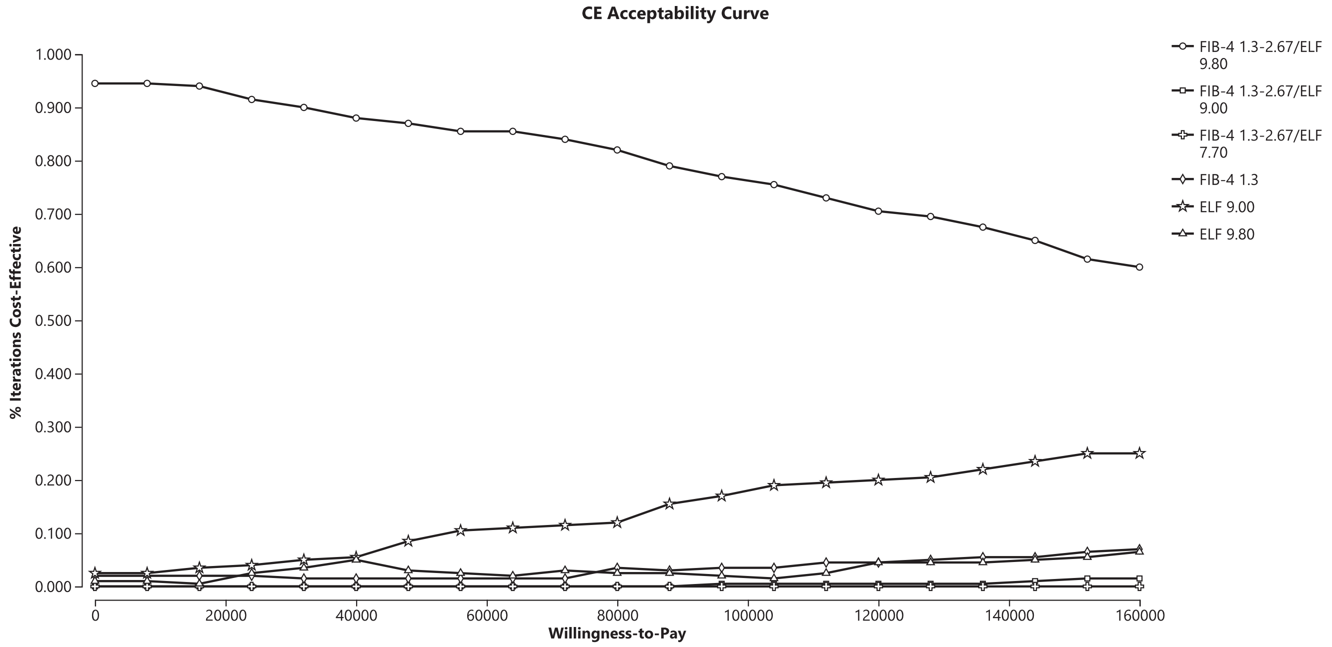

Figure 3. Probability sensitivity analysis for a cost-effectiveness analysis of blood-based fibrosis screening in a real-world population with suspected MASLD: the subgroup aged ≥65. This plot displays, for the subgroup aged ≥65 years, the probability that each strategy was the most cost-effective (y-axis) as willingness-to-pay (x-axis) increased from \$0 to \$160,000/QALY. ELF, enhanced liver fibrosis; FIB-4, fibrosis-4; MASLD, metabolic dysfunction–associated steatotic liver disease; NIT, noninvasive test.

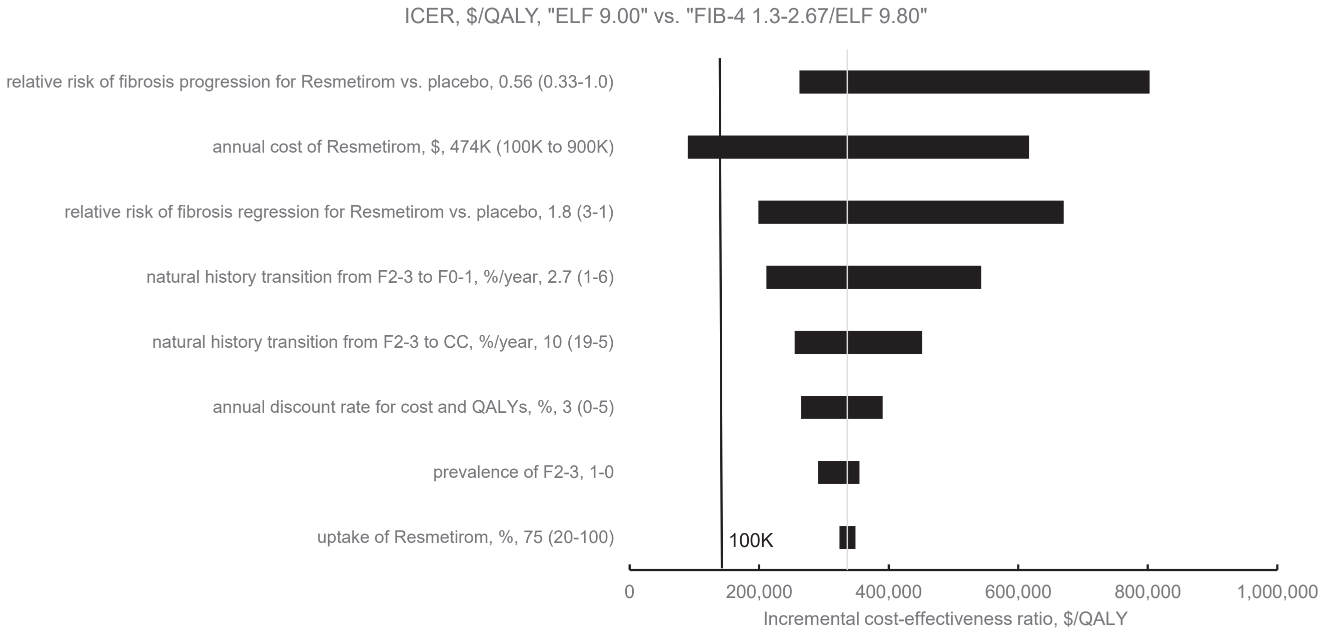

Figure 4. One-way sensitivity analysis of the ICER between the strategies "ELF 9.00" and "FIB-4 1.3–2.67/ELF 9.80." This tornado diagram displays the impact of varying individual parameters on the change in ICER between annual screening using ELF at a 9.00 cutoff ("ELF 9.00") and annual screening using ELF at a 9.80 cutoff to follow indeterminate FIB-4 ("FIB-4 1.3–2.67/ELF 9.80"). For each parameter, the base case value is listed first, followed by the range in the parentheses. The base case values led to the thin vertical lines through the diagram, where the ICER was $336,050/QALY. The left value in the parentheses led to the lower ICER from the sensitivity analysis and the right value led to the higher ICER. A longer bar reflects a greater change in ICER as the parameter was varied. The black thick line at $100,000/QALY marks the willingness-to-pay threshold. The bars that extend to the right of this line mark where "ELF 9.00" was the most cost-effective compared to "FIB-4 1.3–2.67/ELF 9.80," while the bars that extend to the left mark where "FIB-4 1.3–2.67/ELF 9.80" was more cost-effective than "ELF 9.00." ELF, enhanced liver fibrosis; F2-3, fibrosis stage 2-3; FIB-4, fibrosis-4; ICER, incremental cost-effectiveness ratio; QALY, quality-adjusted life-years.

growing plurality of simulations. The strategy "FIB-4 1.3" was likely more cost-effective with higher thresholds in the full population but not among an older cohort (Figure 3).

One-Way Sensitivity Analysis

The parameter most influential on the ICER between "ELF 9.00" and "FIB-4 1.3–2.67/ELF 9.80" was Resmetirom cost: varying from $10,000 to $90,000/y increased the ICER from $89,660/QALY to $616,690/QALY (Figure 4). At a $100,000/QALY willingness-to-pay threshold, the threshold cost of Resmetirom was $11,570/y (76% reduction from base case at $47,400/y), below which "ELF 9.00" would replace "FIB-4 1.3–2.67/ELF 9.80" as the most cost-effective strategy. Treatment effect was influential on the ICER but did not alter this conclusion; for example, even if Resmetirom reduced fibrosis progression risk 3-fold or increased fibrosis regression risk 3-fold, both compared to no treatment, the ICER remained above $100,000/QALY, leaving "FIB-4 1.3–2.67/ELF 9.80" as most cost-effective. Other parameters that impacted the ICER but also did not change this conclusion included the following: the effect of Resmetirom on fibrosis regression, transition probability from F2-3 to F0-1, transition from F2-3 to CC, discount rate for cost and QALYs, and treatment uptake.

Discussion

We evaluated the clinical and cost-effectiveness of 6 blood-based NIT strategies to risk stratify an empiric population with suspected MASLD, explicitly linking real-world diagnostic test performance to contemporary treatment of F2-3 fibrosis with Resmetirom. We found that a 2-tier annual NIT strategy using ELF (cutoff 9.80) following indeterminate FIB-4 (1.3–2.67) ("FIB-4 1.3–2.67/ELF 9.8") was the most cost-effective at a willingness-to-pay threshold of $100,000/QALY. The strategy "FIB-4 1.3–2.67/ELF 9.80" remained optimal across wide range of sensitivity analyses, including scenarios in which treatment costs were reduced by up to 76% and when screening was applied to populations with fewer metabolic comorbidities or older age distributions, where diagnostic accuracy may be confounded by age. Collectively, these findings highlight how integrating practical, scalable NIT-based pathways with emerging therapies can inform guideline implementation and improve early detection in general primary care populations.

Other NIT strategies offered modest clinical benefit by enabling earlier detection and treatment but incurred substantially higher staging and drug costs, limiting their cost-effectiveness. In probability sensitivity analysis, single-tier

NITs—FIB-4 at a 1.3 cutoff ("FIB-4 1.3") and ELF at a 9.00 cutoff ("ELF 9.00")—gained some dominance at higher willingness-to-pay but remained less favorable than "FIB-4 1.3–2.67/ELF 9.80." In older patients, however, declining sensitivity of FIB-4-based strategies had reduced clinical value, leaving only "ELF 9.00" as likely cost-effective besides "FIB4 1.3–2.67/ELF 9.80." Notably, "ELF 9.00" maintained 100% sensitivity across age groups, ensuring all eligible individuals were treated early, and yielded greater LE gains than "FIB-4 1.3–2.67/ELF 9.80" in those aged ≥65 years and those with lower prevalence of T2D or obesity.

One-way sensitivity analysis revealed that Resmetirom cost was most influential and the only input that altered strategy dominance. Specifically, if Resmetirom cost fell below $11,570/y (76% reduction from base case, $47,400/y), "ELF 9.00" would become more cost-effective than "FIB-4 1.3–2.67/ELF 9.80." Compared to "FIB-4 1.3–2.67/ELF 9.80," higher sensitivity of "ELF 9.00" improved long-term clinical outcomes through treating 3–4 more patients per 100. However, its lower specificity led to more unnecessary referrals and higher cost primarily driven by Resmetirom. This suggests that reduced drug cost is needed to support adoption of single-tier NITs by enabling cost-effective treatment. Several US-based studies,[46,51,52] including one by the Institute for Clinical and Economic Review and another by the drug manufacturer, found treating all with significant fibrosis cost-effective at $100,000/QALY with Resmetirom priced between $15,410 and 26,280/y—well below the current $47,400/y as our threshold of $11,570/y for "ELF 9.00" to outperform "FIB-4/ELF 9.80." While our comparison was not between treatment and no treatment but between different NIT strategies, the improvement in clinical outcomes was driven by greater treatment uptake, with costs dominated by drug price. Thus, the ICERs across NITs paralleled treatment vs no treatment. Our finding reinforces that moderate Resmetirom pricing both increases treatment value and expands cost-effective screening options. Ultimately, drug price is the main determinant of long-term screening cost-effectiveness.

Multiple studies assessed cost-effectiveness of NIT-based screening assuming nonpharmacological interventions as the downstream impact.[30,31,53] To our knowledge, our study is among the first to incorporate Resmetirom, a major strength.[32] One prior study compared NIT strategies with no screening and found FIB-4 followed by TE or ELF (9.80 cutoff) most cost-effective (ICERs < $50,000/QALY) even at $47,400/y for Resmetirom.[32] Currently in most settings, TE requires a referral to hepatology. Though we employed similar model structures for the MASLD natural history progression, the study assumed benefit from Resmetirom at F1, whereas we limited treatment effect to F2-3 per its approved use.[40] This more conservative approach likely explains the difference between our ICER findings. Another primary strength of our study lies in the use of real-world patient data on test performance. This allowed us to extrapolate, with confidence, the long-term value of NITs in risk stratifying a population with suspected MASLD. In a few short-term economic evaluations,[27–29] including one based on a preliminary sample of our patient data, "FIB-4 1.3–2.67/ELF 9.80" was identified as the most cost-effective NIT strategy due to minimized unnecessary referrals. Our study extends this discovery by showing that "FIB-4 1.3–2.67/ELF 9.80" remained the most long-term cost-effective NIT strategy across wide sensitivity analysis ranges.

Our findings align with the evolving landscape of MASLD burden and clinical practice. Recent studies from Japan,[18] US,[16,54] and Canada[17] have reported increasing liver stiffness measurements at the national level using TE-based criteria, reflecting growing clinical recognition of advanced fibrosis and expanding real-world application of fibrosis assessment. In this broader context, our results are particularly timely: as demand for and capacity to perform fibrosis evaluation increase, scalable and accessible screening programs are needed upstream of specialist care. Blood-based NITs such as FIB-4 and ELF, both readily available in primary care, can serve as cost-effective triage tools to identify individuals most likely to benefit from specialist referral and effective pharmacological therapies, complementing downstream imaging-based fibrosis assessment.

Globally, reducing barriers to screening implementation and primary care integration remain pressing issues, further underscoring the relevance of our findings. In the United States, efforts to improve MASLD screening in primary care include the development and testing of multicomponent care pathways incorporating electronic health record-based triggers to accelerate specialist referrals,[13] as well as pilot educational interventions aimed at increasing provider awareness and guideline adherence.[55] In parallel, the Nara Declaration and related analyses have emphasized elevated alanine ALT (ALT > 30 U/L) as a straightforward, automated upstream trigger for primary care-based risk stratification of liver fibrosis.[14,15] While ALT alone lacks sufficient specificity for fibrosis staging, its growing recognition as an informative initial filter highlights the need for downstream noninvasive risk stratification tools that are feasible in primary care. In this setting, our proposed sequential screening strategy using FIB-4 and ELF provides a scalable and pragmatic framework for refining fibrosis risk after initial identification, aligning naturally with this growing stream of primary care-focused initiative.

Our study has several limitations. First, we restricted screening impact to Resmetirom use and did not model other potential MASLD treatment options such as Semaglutide (Wegovy). Due to the recency of Wegovy's FDA approval, it is unclear how it will be used as an alternative or simultaneous MASH therapy to Resmetirom. In addition, we assumed that different screening approaches would not lead to additional use of Wegovy, given that it is likely already prescribed for our modeled cohort with T2D and/or obesity. Second, there is paucity of data on the real-world adoption of and persistence with Resmetirom. We

varied both uptake and discontinuation rates widely in sensitivity analyses and found neither to be influential on key conclusions. Third, we modeled treatment efficacy as constant over time based on 52-week clinical trial results. It is uncertain how Resmetirom can sustain fibrosis improvement in the long-term. Our sensitivity analyses showed that even with much higher efficacy, the preferred strategy would remain unchanged. Fourth, our modeled population was derived from a real-world veteran cohort that was predominantly older, with high prevalence of T2D and obesity, which may limit generalizability to other populations. However, scenario analysis focusing on a population with fewer metabolic comorbidities and subgroup analysis incorporating the age impact on screening performance confirmed that our key conclusions remained robust. Finally, we did not include imaging modalities such as TE in our comparisons because our focus is on screening in primary care with limited TE availability.

In this study, we did not consider the health equity impact of blood-based NIT strategies. Populations differing in socioeconomic status, for example, might benefit unequally from screening approaches due to varying disease prevalence and access to care. Future studies should investigate the distributional cost-effectiveness of these NIT strategies for different socioeconomic subpopulations.

In the new therapeutic era of MASLD, sequential testing with indeterminate FIB-4 (1.3–2.67) followed by ELF at a 9.80 cutoff represents the most cost-effective noninvasive screening strategy for primary care. While single-tier ELF screening at a 9.00 cutoff maximized clinical benefit through identifying all treatment-eligible individuals, it also increased unnecessary referrals and is economically justifiable only if drug costs are lower. Overall, these results support broader adoption of blood-based NIT strategies in both high-risk and general primary care populations. As new MASLD therapies emerge and guidelines evolve, ongoing evaluation of the comparative cost-effectiveness of screening approaches will be essential to optimize both clinical outcomes and health-care value.

Conclusion

Sequential testing with ELF at a 9.80 cutoff to follow indeterminate FIB-4 represents a cost-effective, noninvasive MASLD fibrosis screening strategy in primary care.

Supplementary Materials

Material associated with this article can be found, in the online version, at https://doi.org/10.1016/j.gastha.2026.100923.

References

1. Le MH, Yeo YH, Zou B, et al. Forecasted 2040 global prevalence of nonalcoholic fatty liver disease using hierarchical bayesian approach. Clin Mol Hepatol 2022;28(4):841–850.
2. Younossi ZM, Stepanova M, Ong J, et al. Nonalcoholic steatohepatitis is the most rapidly increasing indication for liver transplantation in the United States. Clin Gastroenterol Hepatol 2021;19(3):580–589.e5.
3. Desai R, Jiang Y, VanWagner LB, et al. Financial burden in a US cohort of patients with HCC. Hepatol Commun 2024;8(6):e0453.
4. Lee DU, Hastie DJ, Lee KJ, et al. The trends in cost associated with liver transplantation in the US: analysis of weighted hospital data. Liver Transpl 2023;29(6):626–643.
5. Kim D, Loomba R, Ahmed A. Current burden of MASLD, MetALD, and hepatic fibrosis among US adults with prediabetes and diabetes, 2017-2023. Clin Mol Hepatol 2025;31:e235–e238.
6. Barb D, Repetto EM, Stokes ME, et al. Type 2 diabetes mellitus increases the risk of hepatic fibrosis in individuals with obesity and nonalcoholic fatty liver disease. Obesity (Silver Spring) 2021;29(11):1950–1960.
7. Nyberg LM, Cheetham TC, Patton HM, et al. The natural history of NAFLD, a community-based study at a large health care delivery system in the United States. Hepatol Commun 2021;5(1):83–96.
8. Stefan N, Yki-Järvinen H, Neuschwander-Tetri BA. Metabolic dysfunction-associated steatotic liver disease: heterogeneous pathomechanisms and effectiveness of metabolism-based treatment. Lancet Diabetes Endocrinol 2025;13(2):134–148.
9. Chhatwal J, Dalgic OO, Chen W, et al. Analysis of a simulation model to estimate long-term outcomes in patients with nonalcoholic fatty liver disease. JAMA Netw Open 2022;5(9):e2230426.
10. Palladino A, Gee M, Shalhoub V, et al. Analytical performance of the Enhanced Liver Fibrosis (ELF) Test on the Atellica IM Analyzer. Clin Chim Acta 2023;548:117461.
11. Dawod S, Brown K. Non-invasive testing in metabolic dysfunction-associated steatotic liver disease. Front Med 2024;11:1499013.
12. Serra-Burriel M, Juanola A, Serra-Burriel F, et al. Development, validation, and prognostic evaluation of a risk score for long-term liver-related outcomes in the general population: a multicohort study. Lancet 2023;402(10406):988–996.
13. Godwin KM, Grigoryan L, Thrift AP, et al. A cluster randomized trial of a Multicomponent Clinical Care Pathway (MCCP) to improve MASLD diagnosis and management in primary care: study protocol. BMC Health Serv Res 2025;25(1):645.
14. Kawaguchi T, Yoshiji H, Mochida S, et al. The nara declaration: a new collaborative flow for chronic liver disease between primary care physicians and gastroenterologists/hepatologists. Hepatol Res 2026;56(1):3–8.
15. Nakano M, Kawaguchi M, Kawaguchi T, et al. Profiles associated with significant hepatic fibrosis consisting of alanine aminotransferase >30 U/L, exercise habits, and metabolic dysfunction-associated steatotic liver disease. Hepatol Res 2024;54(7):655–666.
16. Unalp-Arida A, Ruhl CE. Prevalence of metabolic dysfunction-associated steatotic liver disease and fibrosis defined by liver elastography in the United

States using National Health and Nutrition Examination Survey 2017-March 2020 and August 2021-August 2023 data. Hepatology 2025;82(5):1256–1273.
17. Romano J, Burnside J, Sebastiani G, et al. Examining the prevalence of hepatic steatosis and advanced fibrosis using non-invasive measures across Canada: a national estimate using the Canadian Health Measures Survey (CHMS) from 2009-2019. Ann Hepatol 2025; 30(1):101757.
18. Nakano M, Kawaguchi M, Kuwaki K, et al. Nationwide trends in liver stiffness measurement in Japan: a real-world evidence of NILDA using the national database of health insurance claims. Hepatol Res 2026; 56:387–391.
19. Siemens' enhanced liver fibrosis test gets FDA breakthrough device designation. https://clpmag.com/disease-states/diabetes-metabolic/siemens-enhanced-liver-fibrosis-test-gets-fda-breakthrough-device-designation/. Accessed October 21, 2025.
20. European Association for the Study of the Liver (EASL), European Association for the Study of Diabetes (EASD), European Association for the Study of Obesity (EASO). EASL-EASD-EASO Clinical Practice Guidelines on the management of metabolic dysfunction-associated steatotic liver disease (MASLD). J Hepatol 2024; 81(3):492–542.
21. Cusi K, Isaacs S, Barb D, et al. American Association of Clinical Endocrinology Clinical Practice Guideline for the diagnosis and management of Nonalcoholic Fatty Liver Disease in primary care and endocrinology clinical settings: co-sponsored by the American Association for the Study of Liver Diseases (AASLD). Endocr Pract 2022; 28(5):528–562.
22. FDA approves first treatment for patients with liver scarring due to fatty liver disease. https://www.fda.gov/news-events/press-announcements/fda-approves-first-treatment-patients-liver-scarring-due-fatty-liver-disease. Accessed October 21, 2025.
23. FDA approves treatment for serious liver disease known as 'MASH'. https://www.fda.gov/drugs/news-events-human-drugs/fda-approves-treatment-serious-liver-disease-known-mash. Accessed October 21, 2025.
24. Ratziu V, Tacke F. At the dawn of potent therapeutics for fatty liver disease - introducing the miniseries on promising pharmacological targets for NASH. J Hepatol 2023;79(2):261–262.
25. Lomonaco R, Godinez Leiva E, Bril F, et al. Advanced liver fibrosis is common in patients with type 2 diabetes followed in the outpatient setting: the need for systematic screening. Diabetes Care 2021;44(2):399–406.
26. Tsochatzis EA, Valenti L, Thiele M, et al. Use of non-invasive diagnostic tools for metabolic dysfunction-associated steatohepatitis: a qualitative exploration of challenges and barriers. Liver Int 2024;44(8):1990–2001.
27. Pussinen C, Kelkka A, Pulkki K, et al. Enhanced liver fibrosis test improves diagnostic efficiency and reduces testing costs for advanced liver fibrosis in steatotic liver disease. Scand J Gastroenterol 2025;60:728–736.
28. Srivastava A, Jong S, Gola A, et al. Cost-comparison analysis of FIB-4, ELF and fibroscan in community pathways for non-alcoholic fatty liver disease. BMC Gastroenterol 2019;19(1):122.
29. Yeramaneni S, Chang ST, Cheung RC, et al. Comparison of referral rates and costs using Fibrosis-4 and Enhanced Liver Fibrosis (ELF) testing strategies for initial evaluation of metabolic dysfunction-associated steatotic liver disease (MASLD) in a veteran population. J Appl Lab Med 2025;10(3):593–604.
30. Gruneau L, Kechagias S, Sandström P, et al. Cost-effectiveness analysis of noninvasive tests to identify advanced fibrosis in non-alcoholic fatty liver disease. Hepatol Commun 2023;7(7):e00191.
31. Noureddin M, Jones C, Alkhouri N, et al. Screening for nonalcoholic fatty liver disease in persons with type 2 diabetes in the United States is cost-effective: a comprehensive cost-utility analysis. Gastroenterology 2020;159(5):1985–1987.e4.
32. Younossi ZM, Paik JM, Henry L, et al. Pharmaco-economic assessment of screening strategies for high-risk MASLD in primary care. Liver Int 2025;45(4):e16119.
33. Rinella ME, Neuschwander-Tetri BA, Siddiqui MS, et al. AASLD Practice Guidance on the clinical assessment and management of nonalcoholic fatty liver disease. Hepatology 2023;77(5):1797–1835.
34. Sanders GD, Neumann PJ, Basu A, et al. Recommendations for conduct, methodological practices, and reporting of cost-effectiveness analyses: second panel on cost-effectiveness in health and medicine. JAMA 2016;316(10):1093–1103.
35. Husereau D, Drummond M, Augustovski F, et al. Consolidated Health Economic Evaluation Reporting Standards 2022 (CHEERS 2022) statement: updated reporting guidance for health economic evaluations. BMC Med 2022;20(1):23.
36. Products - life tables - homepage. https://www.cdc.gov/nchs/products/life_tables.htm. Accessed October 21, 2025.
37. Kwong AJ, Kim WR, Lake JR, et al. OPTN/SRTR 2022 annual data report: liver. Am J Transplant 2024; 24(2S1):S176–S265.
38. Madrigal pharmaceuticals, Inc. A phase 3, multinational, double-blind, randomized, placebo-controlled study of MGL-3196 (resmetirom) in patients with non-alcoholic steatohepatitis (NASH) and fibrosis to resolve NASH and reduce progression to cirrhosis and hepatic decompensation. clinicaltrials.gov. 2024. https://clinicaltrials.gov/study/NCT03900429. Accessed June 25, 2025.
39. Shuaibi S, Tobal I, Gore J, et al. A real-world experience with resmetirom: tolerability and access. Gastro Hep Adv 2025;4(9):100709.
40. Harrison SA, Bedossa P, Guy CD, et al. A phase 3, randomized, controlled trial of Resmetirom in NASH with liver fibrosis. N Engl J Med 2024;390(6):497–509.
41. Younossi ZM, Tampi R, Priyadarshini M, et al. Burden of illness and economic model for patients with nonalcoholic steatohepatitis in the United States. Hepatology 2019;69(2):564–572.
42. Younossi ZM, Blissett D, Blissett R, et al. The economic and clinical burden of nonalcoholic fatty liver disease in the United States and Europe. Hepatology 2016; 64(5):1577–1586.
43. Sanyal AJ, Banas C, Sargeant C, et al. Similarities and differences in outcomes of cirrhosis due to nonalcoholic

steatohepatitis and hepatitis C. Hepatology 2006;43(4):682–689.
44. Orci LA, Sanduzzi-Zamparelli M, Caballol B, et al. Incidence of hepatocellular carcinoma in patients with nonalcoholic fatty liver disease: a systematic review, meta-analysis, and meta-regression. Clin Gastroenterol Hepatol 2022;20(2):283–292.e10.
45. Mohan BP, Iriana S, Khan SR, et al. Outcomes of liver transplantation in patients 70 years or older: a systematic review and meta-analysis. Ann Hepatol 2022;27(6):100741.
46. Le P, Dasarathy S, Herman WH, et al. Value-based pricing of resmetirom for metabolic dysfunction-associated steatotic liver disease. JAMA Netw Open 2025;8(6):e2517122.
47. Kaplan DE, Chapko MK, Mehta R, et al. Healthcare costs related to treatment of hepatocellular carcinoma among veterans with cirrhosis in the United States. Clin Gastroenterol Hepatol 2018;16(1):106–114.e5.
48. Prescription prices, coupons & pharmacy information. GoodRx. https://www.goodrx.com. Accessed November 3, 2025.
49. Briggs AH, Weinstein MC, Fenwick EAL, et al. Model parameter estimation and uncertainty: a report of the ISPOR-SMDM Modeling Good Research Practices Task Force–6. Value Health 2012;15(6):835–842.
50. McPherson S, Hardy T, Dufour JF, et al. Age as a confounding factor for the accurate non-invasive diagnosis of advanced NAFLD fibrosis. Am J Gastroenterol 2017;112(5):740–751.
51. Non-alcoholic steatohepatitis. ICER. https://icer.org/assessment/non-alcoholic-steatohepatitis-2023/. Accessed October 21, 2025.
52. Javanbakht M, Fishman J, Moloney E, et al. Early cost-effectiveness and price threshold analyses of resmetirom: an investigational treatment for management of nonalcoholic steatohepatitis. Pharmacoecon Open 2022;7(1):93–110.
53. Sangha K, Chang ST, Cheung R, et al. Cost-effectiveness of MRE versus VCTE in staging fibrosis for nonalcoholic fatty liver disease (NAFLD) patients with advanced fibrosis. Hepatology 2023;77(5):1702–1711.
54. van Kleef LA, Pustjens J, Janssen HLA, et al. Diagnostic accuracy of the LiverRisk score to detect increased liver stiffness among a United States general population and subgroups. J Clin Exp Hepatol 2025;15(4):102512.
55. Kumar S, Mohanty A, Mantry P, et al. Deploying a metabolic dysfunction-associated steatohepatitis consensus care pathway: findings from an educational pilot in three health systems. BMC Prim Care 2024;25(1):265.

Received November 23, 2025. Accepted March 9, 2026.

Correspondence:
Address correspondence to: Wanyi Chen, PhD, Siemens Healthcare Diagnostics Inc, 511 Benedict Ave, Tarrytown, New York 10591. e-mail: wanyi.chen@siemens-healthineers.com.

Authors' Contributions:
Wanyi Chen: Study design, data analysis, interpretation of results, and drafting the article. Stephanie T. Chang: Study design, and interpretation of results. Ramsey C. Cheung: Study design, and interpretation of results. Donald B. Chalfin: Interpretation of results. Kinpritma Sangha: Interpretation of results. Szu-Yu Zoe Kao: Interpretation of results. Artem T. Boltyenkov: Study design, interpretation of results, and drafting the article. All authors: Critical revision of the article and final approval of submitted version.

Conflicts of Interest:
The authors disclose the following: Wanyi Chen, Donald B. Chalfin, Kinpritma Sangha, Szu-Yu Zoe Kao, and Artem T. Boltyenkov are employees of Siemens Healthineers. Stephanie Change and Ramsey Cheung received research grants from Siemens Healthineers.

Funding:
This study was funded by Siemens Healthcare Diagnostics Inc, Tarrytown, NY, USA who contributed to conceptualization, design, data collection, analysis, decision to publish, and preparation of the article.

Ethical Statement:
This study is a computer-based modeling study using only de-identified, aggregated, and publicly available data sources. No human subjects were recruited, and no individually identifiable patient information was accessed. Therefore, institutional review board approval was not required.

Data Transparency Statement:
The data used in the preparation of this scientific article were obtained from a third-party source and are jointly owned by VA Palo Alto and Siemens Medical Solutions. Siemens Healthineers does not claim exclusive ownership of this data and fully acknowledges VA Palo Alto and Siemens Medical Solutions as the rightful owners. The use of this data in this article was conducted in accordance with all applicable agreements and permissions, ensuring compliance with institutional and regulatory requirements. The model used to generate the findings of this study is available from the corresponding author upon reasonable request and will be shared via email for noncommercial research purposes.

Reporting Guidelines:
Consolidated Health Economic Evaluation Reporting Standards,[1] with a compliance checklist provided in Supplementary Appendix.

Gastro Hep Advances, Volume 5

Supplemental information

Cost-Effectiveness of Blood-Based Fibrosis Screening in High-Risk Metabolic Liver Diseases With Emerging Therapies

Wanyi Chen, Stephanie T. Chang, Ramsey C. Cheung, Donald B. Chalfin, Kinpritma Sangha, Szu-Yu Zoe Kao, and Artem T. Boltyenkov

Supplementary Appendix

Cost-effectiveness of blood-based fibrosis screening in high-risk metabolic liver diseases with emerging therapies

Wanyi Chen[1], Stephanie T. Chang[2,3], Ramsey C. Cheung[4,5], Donald B. Chalfin[1,6], Kinpritma Sangha[7], Szu-Yu Zoe Kao[7], Artem T. Boltyenkov[1]

[1] Medical Affairs, Siemens Healthcare Diagnostic Inc., Tarrytown, NY, US

[2] Department of Radiology, Veterans Affairs Palo Alto Healthcare System, Palo Alto, CA, US

[3] Department of Radiology, Stanford University Medical Center, Stanford, CA, US

[4] Department of Gastroenterology and Hepatology, VA Palo Alto Healthcare System, Palo Alto, CA, US

[5] Department of Gastroenterology and Hepatology, Stanford University Medical Center, Stanford, CA, US

[6] Jefferson College of Population Health, Thomas Jefferson University, Philadelphia, PA, US

[7] Siemens Medical Solutions USA Inc., Malvern, PA, US

Supplementary Introduction

In this supplementary appendix, we provide further details of the cost-effectiveness analysis framework. We also provide additional results.

Supplementary Methods

Cost-effectiveness analysis framework

Cost-effectiveness analysis (CEA) is a well-established analytic framework used to compare the costs and health outcomes of one or more health interventions.[49,50] A CEA aims to identify the health intervention(s) that maximize health benefits subject to resource constraints. Here, we provide a brief survey of the key CEA concepts used in our study.

One of the main outcomes of a CEA is the incremental cost-effectiveness ratio (ICER), defined as the ratio of the difference in costs between two alternative strategies to the difference in effectiveness between the same two alternatives. When performing the base case analysis, strategies are first ordered based on increasing costs. Strategies can be eliminated if they result in lower health benefits (e.g., QALYs) and cost more than an alternative (namely, strong dominance). After eliminating strategies based on strong dominance, the remaining strategies are ordered in both increasing costs and health outcomes. To identify the most cost-effective strategy among the remaining contenders, the concept of weak dominance first applies. Consider three strategies of increasing costs and health outcomes: A, B, and C. Strategy B can be eliminated based on weak dominance if the ICER between C and A is lower than the ICER between B and A. Intuitively, this is because both B and C improves health outcomes compared to A, however, C improves outcomes at a lower cost per unit of improvement than B does, due to having lower ICER.

After applying both strong and weak dominance rules, the remaining strategies are said to be on the cost-efficiency frontier. To identify the optimal strategy given budget constraints, we need the concept of willingness-to-pay threshold (WTP), defined as the dollar amount that an individual or society is willing to pay for the additional per unit of health benefit gained.[51] The WTP threshold makes the ICER actionable for decision making, as it is used to judge whether the health gains offered by a health intervention are sufficiently large relative to the costs for the intervention to be adopted. In our study, we adopt a widely adopted WTP threshold of $100,000/QALY, which was empirically estimated based on the concept of health opportunity cost.[52]

Finally, the most cost-effective strategy given a WTP threshold is the strategy associated with the greatest ICER (compared to the next costly strategy) that does not exceed the WTP threshold. The remaining strategies on the cost-efficiency frontier are said to be less cost-effective. Those eliminated by dominance rules are said to be not cost-effective.

Supplementary Results

Appendix Figure A1. Natural history of MASLD model structure.

Appendix Table A1. Additional base case results for a cost-effectiveness analysis of blood-based fibrosis screening in a real-world population with suspected MASLD, for the full population, the subgroup aged ≥65 years, and the lower-risk population with fewer metabolic comorbidities.

Annual NIT-based screening strategies	Life expectancy, years	Number of DCC, cases/10,000	Number of HCC, cases/10,000	Number of LT, Cases/10,000	Number treated with Resmetirom/100
**Full population**					
FIB-4 1.3-2.67/ELF 9.80	12.775	447	297	45.8	32.2
ELF 9.80	12.776	446	297	45.5	32.2
FIB-4 1.3-2.67/ELF 9.00	12.808	437	288	44.2	34.9
FIB-4 1.3	12.809	437	288	44.0	34.9
FIB-4 1.3-2.67/ELF 7.70	12.809	437	288	44.0	34.9
ELF 9.00	12.818	435	286	43.6	35.7
**Subgroup aged ≥65 years**					
FIB-4 1.3-2.67/ELF 9.80	12.654	486	322	48.7	21.2
FIB-4 1.3	12.654	484	323	49.3	21.2
FIB-4 1.3-2.67/ELF 9.00	12.655	485	323	49.2	21.2
FIB-4 1.3-2.67/ELF 7.70	12.654	484	323	49.3	21.2
ELF 9.80	12.786	443	293	44.6	33.3
ELF 9.00	12.818	435	286	43.5	35.7

Appendix Table A1. Continued.

Annual NIT-based screening strategies	Life expectancy, years	Number of DCC, cases/10,000	Number of HCC, cases/10,000	Number of LT, Cases/10,000	Number treated with Resmetirom/100
**Lower-risk population with fewer metabolic comorbidities**					
FIB-4 1.3-2.67/ELF 9.80	17.960	280	312	99.5	24.7
ELF 9.80	17.960	280	311	99.4	24.7
FIB-4 1.3-2.67/ELF 9.00	17.966	275	306	97.5	25.8
FIB-4 1.3	17.967	275	306	97.5	25.8
FIB-4 1.3-2.67/ELF 7.70	17.967	275	306	97.5	25.8
ELF 9.00	17.970	274	305	97.1	26.1

Abbreviations: DCC, decompensated cirrhosis; ELF, enhanced liver fibrosis; FIB-4, fibrosis index; HCC, hepatocellular carcinoma; LT, liver transplantation.

CHEERS checklist

	Item	Guidance for Reporting	Reported in section
**TITLE**			
Title	1	Identify the study as an economic evaluation and specify the interventions being compared.	Page 1
**ABSTRACT**			
Abstract	2	Provide a structured summary that highlights context, key methods, results and alternative analyses.	Page 4
**INTRODUCTION**			
Background and objectives	3	Give the context for the study, the study question and its practical relevance for decision making in policy or practice.	Page 5-6
**METHODS**			
Health economic analysis plan	4	Indicate whether a health economic analysis plan was developed and where available.	Page 7-8
Study population	5	Describe characteristics of the study population (such as age range, demographics, socioeconomic, or clinical characteristics).	Page 9
Setting and location	6	Provide relevant contextual information that may influence findings.	Page 7-9
Comparators	7	Describe the interventions or strategies being compared and why chosen.	Page 7
Perspective	8	State the perspective(s) adopted by the study and why chosen.	Page 8
Time horizon	9	State the time horizon for the study and why appropriate.	Page 8
Discount rate	10	Report the discount rate(s) and reason chosen.	Page 8
Selection of outcomes	11	Describe what outcomes were used as the measure(s) of benefit(s) and harm(s).	Page 7-8
Measurement of outcomes	12	Describe how outcomes used to capture benefit(s) and harm(s) were measured.	Page 7-8
Valuation of outcomes	13	Describe the population and methods used to measure and value outcomes.	Page 7-10
Measurement and valuation of resources and costs	14	Describe how costs were valued.	Page 8, 10
Currency, price date, and conversion	15	Report the dates of the estimated resource quantities and unit costs, plus the currency and year of conversion.	Page 8
Rationale and description of model	16	If modelling is used, describe in detail and why used. Report if the model is publicly available and where it can be accessed.	Page 8-9
Analytics and assumptions	17	Describe any methods for analysing or statistically transforming data, any extrapolation methods, and approaches for validating any model used.	NA

Characterizing heterogeneity	18	Describe any methods used for estimating how the results of the study vary for sub-groups.	Page 6-7
Characterizing distributional effects	19	Describe how impacts are distributed across different individuals or adjustments made to reflect priority populations.	NA
Characterizing uncertainty	20	Describe methods to characterize any sources of uncertainty in the analysis.	Page 9-11
Approach to engagement with patients and others affected by the study	21	Describe any approaches to engage patients or service recipients, the general public, communities, or stakeholders (e.g., clinicians or payers) in the design of the study.	NA
**RESULTS**			
Study parameters	22	Report all analytic inputs (e.g., values, ranges, references) including uncertainty or distributional assumptions.	Page 9-10, Table 1 and 2
Summary of main results	23	Report the mean values for the main categories of costs and outcomes of interest and summarise them in the most appropriate overall measure.	Page 12-14, Table 3, Figure 1-3
Effect of uncertainty	24	Describe how uncertainty about analytic judgments, inputs, or projections affect findings. Report the effect of choice of discount rate and time horizon, if applicable.	Page 13-14, Figure 2-3
Effect of engagement with patients and others affected by the study	25	Report on any difference patient/service recipient, general public, community, or stakeholder involvement made to the approach or findings of the study	NA
**DISCUSSION**			
Study findings, limitations, generalizability, and current knowledge	26	Report key findings, limitations, ethical or equity considerations not captured, and how these could impact patients, policy, or practice.	Page 15-17
**OTHER RELEVANT INFORMATION**			
Source of funding	27	Describe how the study was funded and any role of the funder in the identification, design, conduct, and reporting of the analysis	Page 2
Conflicts of interest	28	Report authors conflicts of interest according to journal or International Committee of Medical Journal Editors requirements.	Page 2

Natural history model

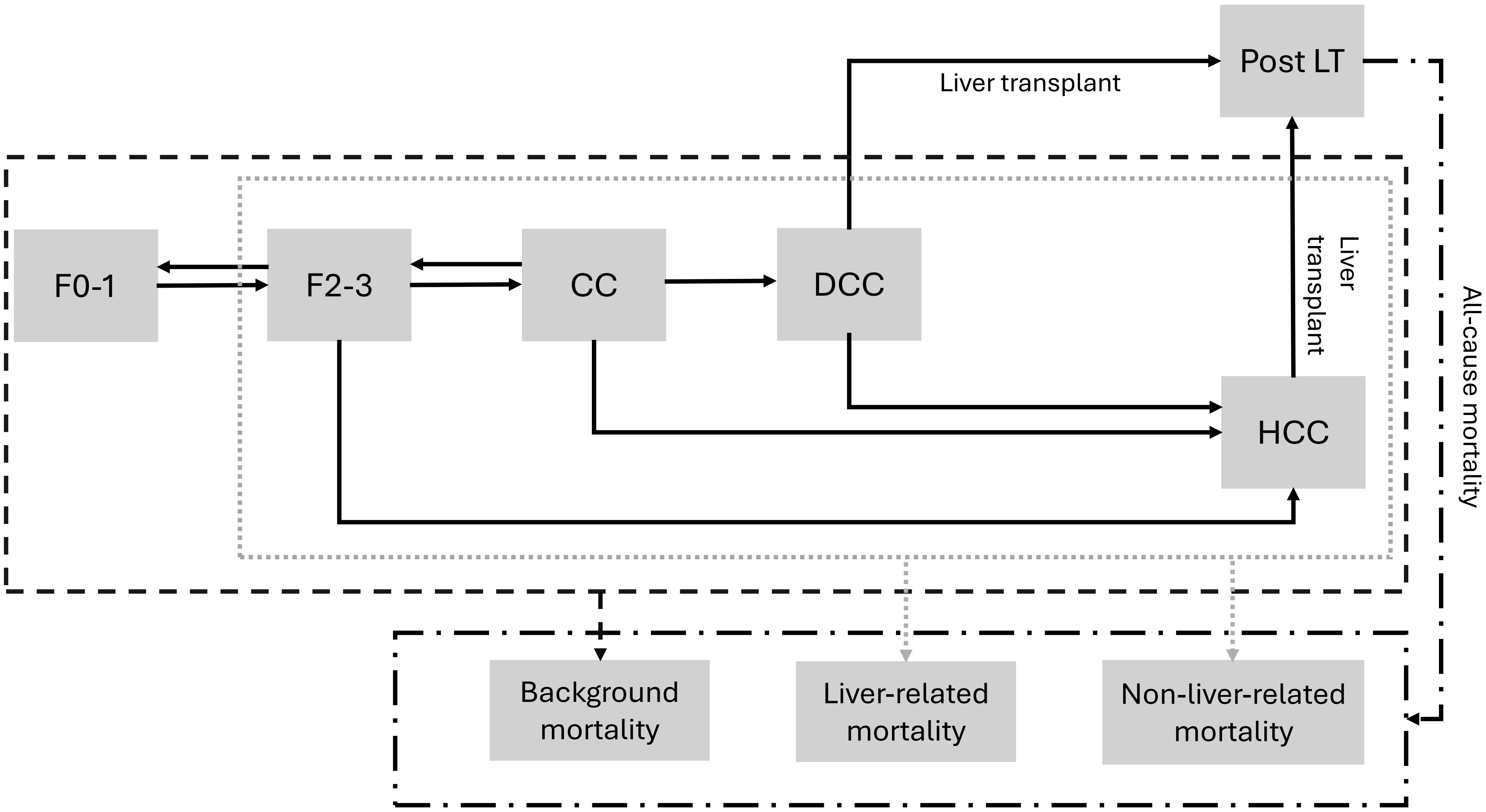

Supplement: Extended PDF [file mmc3.pdf]
